# Left atrial appendage aneurysm in pediatrics: Case study and literature review

**DOI:** 10.3389/fcvm.2023.1211619

**Published:** 2023-08-10

**Authors:** Kambiz Norozi, Mathushan Subasri, Luis Altamirano Diaz, Osami Honjo

**Affiliations:** ^1^ Department of Paediatrics, University of Western Ontario, London, ON, Canada; ^2^ Division of Pediatric Cardiology, Department of Paediatrics, London Health Sciences Centre, London, ON, Canada; ^3^ Pediatric Cardiopulmonary Research Laboratory, London Health Sciences Centre, London, ON, Canada; ^4^ Children Health Research Institute, London, ON, Canada; ^5^ Department of Paediatric Cardiology, Medical School Hannover, Hannover, Germany; ^6^ Faculty of Medicine and Health Sciences, McGill University, Montreal, QC, Canada; ^7^ Department of Surgery, The Hospital for Sick Children, Toronto, ON, Canada

**Keywords:** left atrial appendage aneurysm, pediatric cardiology, pediatric cardiac surgery, case report, literature review

## Abstract

Left atrial appendage aneurysm (LAAA) is a rare cardiac pathology that is often identified in adulthood. There are a myriad of presentations related to atrial appendage enlargement, but most are asymptomatic. Pediatric cases of LAAA are extremely rare. We report a case of an incidental giant LAAA found in a healthy 6-year-old boy. He was successfully treated with surgical resection. A review of the literature shows that the presentation of LAAA in pediatrics likely involves cardiac or respiratory symptoms but can also be incidental findings. Similar to adults, diagnosis requires cardiac imaging, with echocardiography being the mainstay. Surgical intervention is indicated in symptomatic and most asymptomatic patients to prevent complications. More research is warranted into the optimal timing of surgery and alternative surgical approaches for complex cases.

## Introduction

The left atrial appendage has highly variable morphology and limited known functions. Namely, hemodynamic control via heightened distensibility and stretch mechanoreceptors (1). On rare occasions, a pathological dilation of the appendage can occur. This cardiac pathology is known as the left atrial appendage aneurysm (LAAA) (2, 3). An LAAA can be intrapericardial or extra-pericardial, the latter a result of pericardial defects (4). The etiology of intra-pericardial LAAAs has been organized as either congenital dysplasia of the atrial muscle or acquired from increased left atrial pressure due to mitral valve disease (3, 5). If untreated, it can manifest fatal complications such as tachyarrhythmias, outflow tract obstructions and thromboembolisms (5, 6). Typically, LAAA presents around the third decade of life, owing to progressive aneurysmal growth; as such, pediatric presentations are rare (7–9). Herein we present a case of LAAA in a six-year-old boy successfully treated with resection. In addition, we summarize the existing pediatric LAAA literature to compare presentation, diagnosis, and treatment between children and adults.

## Case presentation

**Figure 1:**
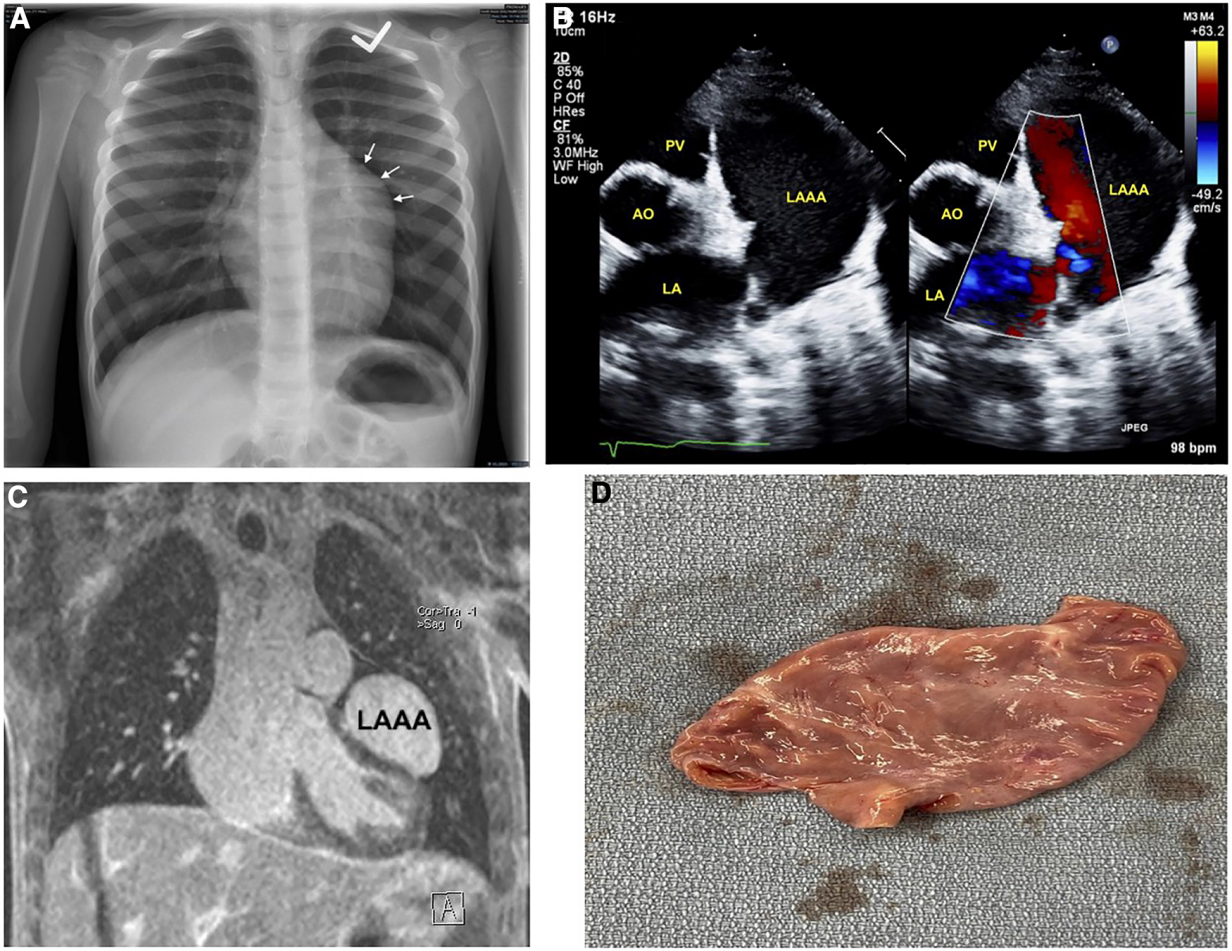
Imaging of left atrial appendage aneurysm. (**A**) Initial chest x-ray showing an unusual contour along the left heart border. (**B**) Transthoracic echocardiography short-axis sweep with colour doppler demonstrating a cavity adjacent to and potentially communicating with the left atrium. (**C**) Magnetic resonance imaging revealing the LAAA, and compressing the left ventricular anterior and anteroseptal walls. (**D**) Post-operative image of the resected LAAA measuring 5.9 × 3.1 × 1.9 cm. AO, aorta; LA, left atrium; LAAA, left atrial appendage aneurysm; PV, pulmonary valve.

An otherwise healthy 6-year-old boy was referred to pediatric cardiology due to an incidentally discovered abnormal cardiac silhouette on a chest x-ray performed during an investigation of scarlet fever with cough (Figure 1A). On history, he was active, able to keep up with his peers, and had no history of cyanosis, syncope, chest pain, or palpitations. There was no family history of congenital cardiac anomalies or early unexplained deaths. His physical exam revealed a well-appearing boy in no distress; height 128 cm and weight 23.9 kg. A cardiac exam revealed a quiet precordium with no heaves or thrills, regular S1 and normally split S2 with no extra heart sounds or murmurs. Right arm (102/64) and left arm (115/66) blood pressures were normal. There was no hepatomegaly, and the respiratory exam was normal: respiratory rate of 24 and oxygen saturation of 98%.

An electrocardiogram was performed and showed normal sinus rhythm. Subsequent baseline echocardiography showed a giant LAAA with otherwise regular heart structure, size, and function (Figure 1B) (Supplementary Data Sheet S1). This was later confirmed with magnetic resonance imaging, which also showed mild compression of the left ventricular anterior and anteroseptal walls (Figure 1C). Surgical intervention was favoured for prophylaxis against compressive or thrombotic events. He underwent surgical resection without any intraoperative complications.

Post-operatively, he initially had vasoplegia that resolved with a transfusion of one unit of packed cells and norepinephrine. Pathology revealed an appendage measuring 5.9 × 3.1 × 1.9 cm, without blood clots (Figure 1D). His 3-day postoperative course was uneventful. Echocardiography at his first post-operative clinic visit showed the LAAA had been plicated with otherwise normal cardiac structure and function. He remained asymptomatic with a normal physical exam, electrocardiogram, and echocardiography at his two-month, 12-month, and 24-month postoperative follow-ups (Table 1).

**Table 1:** Timeline of events.

Days (D)	Event
D1	Ambulatory visit to pediatric cardiology for incidental identification of LAAA
D85	Admission for surgery
D86	Surgical resection
D88	Discharge from hospital
D98	First (1-week) post-op visit—normal
D140	Second (2-month) post-op visit—normal
D506	Third (1-year) post-op visit—normal
D862	Fourth (2-year) post-op visit—normal

## Discussion

The first reports of LAAA were in the 1960's and since the majority of cases observed and published have been in the adult population (10, 11). To this end, our case presentation adds to a limited body of literature. In addition, we performed a robust literature review of existing pediatric LAAA cases. Literature searches using the terms “left atrial appendage” and “aneurysm” were performed in PubMed, Embase, Scopus, and Web of Science. Cases were screened for presentations under 18 years old.

A total of 76 pediatric LAAA cases were identified (Supplementary Data Sheet S2). The average age at intervention was 5.72 ± 5.69 years, with 25/76 (33%) being one year or younger. Overall, there were slightly more male (37/76) than female (30/76) cases (seven cases were missing this information). Nine cases were detected antenatally using fetal echocardiography. Most fetal presentations were asymptomatic, but four cases had symptoms of respiratory distress. Amongst the remaining 67 patients diagnosed postnatally, 35 cases presented with at least one major cardiac-related symptom, 11 cases with only respiratory symptoms, 8 cases with primarily neurological symptoms, 11 cases that were identified incidentally on imaging, and two cases were missing this information.

Respiratory symptoms were mostly forms of distress or cough/hiccups, likely due to phrenic nerve irritation (12, 13). Neurologic symptoms such as motor deficits or seizures are thought to arise from thromboembolism from the left atrial appendage into the systemic circulation (14–16). From cardiac symptoms, palpitations/arrhythmias and murmurs were most common (Table 2). Age-unrestricted reviews of LAAA have found that palpitations, dyspnea on exertion, and chest pain were the most common (5, 6, 8). In our review of pediatric cases, patients often presented with multiple symptoms; for example, four of seven cases with respiratory distress also had palpitations. The suggested mechanism of these complaints is LAAA-mediated compression of the left coronary artery and its tributaries.

**Table 2:** Primary presenting symptoms of LAAA cases.

Cardiac symptoms^a^	
Arrhythmia/Palpitations	15
Murmur	11
Chest pain	6
Hypertension	1
Pre-syncope/Syncope	6
Cyanosis/Low O2 saturation	3
Non-cardiac symptoms^a^	
Respiratory distress	7
Persistent cough/Hiccups	4
Respiratory infection	8
Neurological	8
Cardiomegaly on imaging for non-cardiac reason	12
Urinary tract symptoms	2

^a^ Patients with multiple symptoms were logged into each symptom category.

The average LAAA size was 6.6 ± 3.6 cm × 4.3 ± 2.0 cm. Communications with the left atrium were cited in 46 (61%) cases with an average neck diameter of 2.0 ± 0.8 cm. Thrombus was not identified or not mentioned in 60 (82%), identified in 10 (13%), and possible (i.e., spontaneous contrast in echocardiography) in 3 (5%) cases. From the 10 cases with thrombus identified, five presented with neurological symptoms. While congenital LAAA is derived from atrial muscle dysplasia, acquired LAAA can be secondary to mitral valve (MV) disease (3, 5). Accordingly, we found 16 (21%) patients cited with MV pathologies.

In terms of clinical investigations, primary investigations included electrocardiograms (*n* = 48), transthoracic (TTE) and/or transesophageal (TEE) echocardiograms (*n* = 66), and chest x-rays (*n* = 53). Other imaging investigations included computed tomography (*n* = 27), magnetic resonance imaging (*n* = 20), or cardiac catheterization/angiography (*n* = 14). There are advantages and disadvantages to each imaging modality. Mainstay imaging often included chest radiographs and echocardiography. TTE is less invasive but limited by a small acoustic window, whereas TEE provides greater acuity (17, 18). Some suggest that cardiac MRI is ideal because it is non-invasive, does not expose patients to contrast or radiation, can help rule out other causes of cardiomegaly like tumours or cystic structures, and provides the anatomical and functional detail required for surgical intervention. Using general anesthesia for younger children and oral sedation for adolescents prior to scanning has been shown to be effective (17, 18).

Surgical intervention via thoracotomy or sternotomy was undertaken in most cases (*n* = 63), where only five had postoperative complications, including pericardial effusion (9), supraventricular tachycardia (19), ventricular dysfunction due to compression from the LAAA that persisted post-surgery (20), postoperative fever and elevated erythrocyte sedimentation rate from thrombosis within a catheter-based closure of an LAAA (15), and progressive growth of residual aneurysm which was left behind because the circumflex coronary travelled across the LAAA (21). There was only one mortality in a patient who developed myopericarditis and suffered cardiac arrest before the opportunity to have surgery. Similar to the adult population, surgery remains the primary treatment option for pediatric LAAA to prevent fatal complications later in life (14, 22–26). The most common surgical approach remains the median sternotomy with or without cardiopulmonary bypass. The left lateral thoracotomy has also been described in pediatrics and benefits from reduced surgery-associated trauma but is contraindicated if a thrombus is present within the aneurysm (27). For those with thrombus but are unable or unwilling to undergo surgery, treatment with anticoagulants is warranted (15, 18). Finally, minimally invasive endoscopic approaches have been successful but are seldom described (28–31). With the widespread use of fetal echocardiography, early diagnosis of neonatal LAAA is increasing (20, 32). Symptomatic neonates have been operated on as early as 1 day old (33). However, for asymptomatic neonates with fetal diagnoses, the question remains what indications should guide the timing of surgery (34–36).

## Limitations

The LAAA is a rare entity and even more so in pediatrics, which creates challenges in determining statistically significant differences between adult and pediatric presentations. This was not attempted in this case study and review but should be further investigated in the future.

## Conclusion

Although LAAA is a rare finding in the pediatric population, it should be included in the list of differential diagnoses if left-sided cardiomegaly is observed. The presentations and clinical courses in pediatrics are complementary to adults and suggest surgical intervention; however, the optimal time for surgery in asymptomatic pediatric patients is unclear. Operative complications are rare, but further research needs to be conducted into the management of cases with anatomical variation and the use of transcatheter or minimally invasive interventions.

## Data Availability

The original contributions presented in the study are included in the article/**Supplementary Material**, further inquiries can be directed to the corresponding author/s.
