# Supplementary material for: Left atrial appendage aneurysm in pediatrics: Case study and literature review
Source: Front Cardiovasc Med. 2023 Aug 10;10:1211619. doi: 10.3389/fcvm.2023.1211619 (PMC10449248; doi:10.3389/fcvm.2023.1211619)
Supplement: Supplementary file 4 [file Datasheet1.docx]

Supplementary Material

Left Atrial Appendage Aneurysm in Pediatrics: Case Study and Literature Review

Kambiz Norozi^*^, Mathushan Subasri, Luis Altamirano Diaz, Osami Honjo

*** Correspondence:** Corresponding Author: Kambiz.Norozi@lhsc.on.ca

# Supplementary A. Echocardiography Videos

Echocardiography of LAAA. These videos have been annotated to denote the relevant heart chambers. Two videos are included:

1. LAAA 3 Chamber View
2. LAAA Short-Axis Colour Comparison
