# Supplementary material for: Left atrial appendage aneurysm in pediatrics: Case study and literature review
Source: Front Cardiovasc Med. 2023 Aug 10;10:1211619. doi: 10.3389/fcvm.2023.1211619 (PMC10449248; doi:10.3389/fcvm.2023.1211619)
Supplement: Supplementary file 5 [file Datasheet2.docx]

Supplementary Material

Left Atrial Appendage Aneurysm in Pediatrics: Case Study and Literature Review

Kambiz Norozi^*^, Mathushan Subasri, Luis Altamirano Diaz, Osami Honjo

*** Correspondence:** Corresponding Author: Kambiz.Norozi@lhsc.on.ca

# Supplementary B. Excel Document.

Excel document of all the papers included in the literature review of pediatric left atrial appendage aneurysms.
